# Exploring the drivers of variation in trophic mismatches: A systematic review of long‐term avian studies

**DOI:** 10.1002/ece3.7346

**Published:** 2021-03-20

**Authors:** Mikhail K. Zhemchuzhnikov, Tom S. L. Versluijs, Thomas K. Lameris, Jeroen Reneerkens, Christiaan Both, Jan A. van Gils

**Affiliations:** ^1^ NIOZ Royal Netherlands Institute for Sea Research Den Burg The Netherlands; ^2^ University of Groningen Groningen The Netherlands

**Keywords:** asynchrony, bird phenology, consumer‐resource interactions, reproductive success, trophic mismatch

## Abstract

1. Many organisms reproduce in seasonal environments, where selection on timing of reproduction is particularly strong as consumers need to synchronize reproduction with the peaked occurrence of their food. When a consumer species changes its phenology at a slower rate than its resources, this may induce a trophic mismatch, that is, offspring growing up after the peak in food availability, potentially leading to reductions in growth and survival. However, there is large variation in the degree of trophic mismatches as well as in its effects on reproductive output.
2. Here, we explore the potential causes for variation in the strength of trophic mismatches in published studies of birds. Specifically, we ask whether the changes in the degree of mismatch that have occurred over time can be explained by a bird's (a) breeding latitude, (b) migration distance, and/or (c) life‐history traits.
3. We found that none of these three factors explain changes in the degree of mismatch over time. Nevertheless, food phenology did advance faster at more northerly latitudes, while shifts in bird phenology did not show a trend with latitude.
4. We argue that the lack of support in our results is attributable to the large variation in the metrics used to describe timing of food availability. We propose a pathway to improve the quantification of trophic mismatches, guided by a more rigorous understanding of links between consumers and their resources.

## Graphical abstract

We explore the potential causes for variation in the strength of trophic mismatches in published studies on mismatches on birds. According to our results, neither (a) breeding latitude, nor (b) migration distance, nor (c) life‐history traits could explain changes in the degree of mismatch over time. We argue that the lack of support in our results is caused by the large variation in the metrics used to describe the timing of food availability.

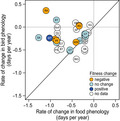

## 1 INTRODUCTION

### 1.1 Global warming produces greater trophic asynchrony

The globe is getting warmer: Land and ocean surface temperatures on the planet have increased by 0.87°C between 1850 and 2015 (IPCC, 2019), and global mean surface temperatures during the last decade (2010–2019) were the warmest on record (WMO, 2020). As a consequence, areas will change dramatically and may be unrecognizable by the end of the 21st century (Overland et al., 2019). Species are forced to adapt to such rapid climate changes (Donnelly et al., 2011), and probably the best‐documented ecological adaptations to climate change are phenological advancements of major life‐history events in annual cycles (Parmesan, 2006; Parmesan & Yohe, 2003; Root et al., 2003; Rosenzweig et al., 2008). Phenological advancements in response to climate change have been shown for many taxonomic groups, including plants (Cleland et al., 2007; Renner & Zohner, 2018), arthropods (Musolin & Saulich, 2012; Robinet & Roques, 2010), amphibians (While & Uller, 2014), birds (Gordo, 2007; Visser et al., 2012), and mammals (Cherry et al., 2013; Moyes et al., 2011). Importantly, rates of advancement differ between taxa (Both et al., 2009; Ge et al., 2015; Høye et al., 2007; Ovaskainen et al., 2013; Root et al., 2003; Thackeray et al., 2010), potentially inducing or increasing so‐called “phenological mismatches,” that is, disruptions of synchronous interactions between ecologically coupled species. Such interactions can either be mutualistic, for instance between plants and pollinators, or antagonistic, usually between consumers and their food source; the latter disruption being coined “trophic mismatch” (Renner & Zohner, 2018). In many cases, food sources tend to advance at faster rates than their consumers (Both et al., 2009). When trophic mismatches result in insufficient food available during the prime period of offspring growth, this can have drastic effects on growth and survival of the offspring, thereby impacting the consumer's fitness (Gaston et al., 2009; Lameris et al., 2018; Ross et al., 2018; Watanuki et al., 2009) and potentially resulting in population declines (Both et al., 2006; Post & Forchhammer, 2008; Saino et al., 2011), but see (Johansson et al., 2015; Miller‐Rushing et al., 2010; Reed et al., 2013). Trophic mismatches during reproduction are therefore considered among the most impactful effects of climate warming on populations (Ockendon et al., 2014), although we still know little about the underlying mechanisms (Kharouba & Wolkovich, 2020).

### 1.2 Expressing a trophic mismatch

To measure the degree of a trophic mismatch, and to be able to make comparisons of mismatches among species and populations, it is necessary to view the advancement in timing of reproduction of the consumer relative to a yardstick which describes the phenology of its main food sources (Visser & Both, 2005). This yardstick most often is timing of the food peak, either expressed in abundance (Corkery et al., 2019; Regular et al., 2014) or quality (Gauthier et al., 2013; Ross et al., 2017, 2018). Besides the timing of the food peak relative to the timing of the consumer's peak demand, a trophic mismatch can be defined relative to other parameters that describe food availability (Box S1). One of them is the “window of opportunity,” which describes the period of sufficient food abundance or quality above a critical threshold based on the individual's maintenance demand (Dunn et al., 2011; Durant et al., 2005, 2007; Reneerkens et al., 2016; Tulp, 2007). However, each yardstick will at its best only be an indicator of the food availability experienced by the consumer. Indicators vary considerably in their precision with which they describe true food availability, and the choice of an indicator will most likely impact whether, and to what extent, study populations are considered trophically mismatched. Moreover, the spatiotemporal resolution at which an indicator is sampled depends on the life history of the focal species, which thus complicates generalizations about trophic mismatches among studies (Kharouba & Wolkovich, 2020).

### 1.3 Fitness consequences of trophic mismatches

In theory, stronger negative impacts on reproductive output are expected when trophic mismatches are larger (Knudsen et al., 2011; Visser et al., 2012). However, large variation in the degree of trophic mismatches and in the (potential) impacts on reproductive output has been reported (Visser et al., 2012). Different measures of reproductive output are used among studies (see Methods), which complicates comparisons.

A number of claims have been made about the causes of the variation in the degree of mismatches (Chmura et al., 2019). For example, the strength of seasonality of habitats or the abundance of alternate prey after the major food peak (Johansson et al., 2015) has been suggested to determine the extent to which bird populations are trophically mismatched. Trophic mismatches typically come about when phenology of the food is advancing more rapidly (or slowly) than phenology of their consumers, because consumers have difficulties to advance (or delay) their reproductive phenology at the same pace (Both et al., 2009). This may be due to time constraints when preparing for reproduction or the inability to predict the optimal timing of reproduction (constraint vs. cues hypothesis (Visser et al., 2012)). Whether a consumer species is able to advance fast enough may be predicted from its life history (McLean et al., 2016). Migratory species, for example, have been suggested to be particularly prone to become trophically mismatched, which was reviewed in detail by (Knudsen et al., 2011), but see (Winkler et al., 2014). Also, trophic mismatches have been suggested to be especially pronounced in the Arctic biome (Post et al., 2009), where climate is changing faster than anywhere else in the world (Box et al., 2019; Post et al., 2018) and food peaks are more pronounced as a result of a short growing season. To the best of our knowledge, these general claims have never been quantitatively tested, most likely because such tests are complicated due to the large variation in the indicators for food availability and because of the correlations between different predictors (e.g., Arctic breeding birds are almost invariably long‐distance migrants). Here, we take up this challenge.

In the current paper, using published long‐term time series of avian species and their food source, we examine (a) rates of change in the degree of trophic mismatches between birds and their food, and (b) whether latitude, migration distance, and life‐history traits can explain variation in the degree of change in trophic mismatches. Based on the outcomes of these analyses, we discuss the complications that arise by using indicators of various precision to describe food availability and the unbalanced nature of existing long‐term datasets.

## 2 METHODS

### 2.1 Search criteria

We queried the Web of Science database (30 April 2019) using the following words and operators in the field TOPIC (search based on title, abstract and key words): (bird* OR avian OR ornitholog*) AND (trophic OR phenolog*) AND (*match* OR *synchron* OR shift* OR snowmelt). The asterisk here is used as replacement for any possible combinations of letters. This resulted in 910 papers. Following (Visser et al., 2012), we subsequently inspected these papers and selected those where food and bird phenology were measured simultaneously for at least 10 years, which has been suggested to be the minimum timespan required to detect trophic mismatches (Miller‐Rushing et al., 2010). In most of the selected studies, food phenology was described using environmental indicators that are shown or expected to be correlated with food phenology (e.g., growing degree days, date of snowmelt, or remotely sensed parameters). In contrast, bird breeding phenology was mostly measured by direct observation (e.g., lay dates, hatch dates, fledge dates). We excluded several studies from our selection because original estimates of phenological shifts were not reported (Dunn et al., 2011; Visser et al., 1998), breeding phenology of birds was indirectly estimated using potentially unreliable indicators such as timing of migration or arrival date (Clausen & Clausen, 2013; Mayor et al., 2017), and studies were conducted in the same area but had partially overlapping study periods with studies spanning a longer time period (Both et al., 2009; Visser et al., 1998).

**FIGURE 1:**
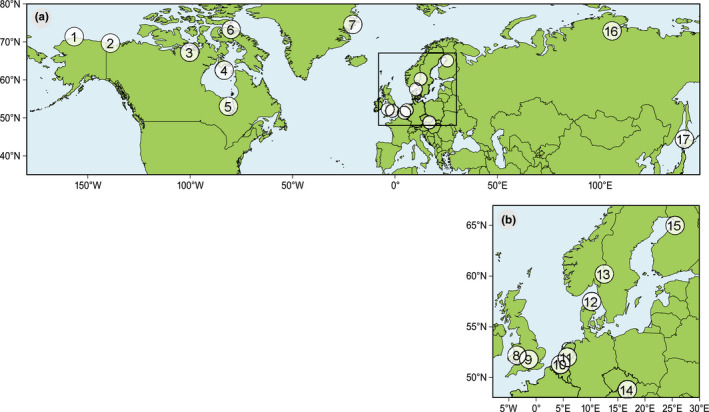
(a) Locations of all study sites included in the analysis. (b) Inset with locations of study sites in Europe. The details of the studies are given in Table 1

If studies included several bird species, and/or several study sites, we included them as separate data points (Bauer et al., 2010; Both et al., 2009; Grabowski et al., 2013; Matthysen et al., 2011; Ross et al., 2017; Saalfeld & Lanctot, 2017; Vatka et al., 2014; Wegge & Rolstad, 2017). Eventually, we used 20 publications with phenological data on 40 populations of 28 bird species and their food (Figure 1, Table 1).

**TABLE 1:** The overview of the study sites on Figure 1

#	Location	Species	Study interval	Number of years	References
1	Utqiagvik	AGP, DU, DO, PS, ReP, RnP, SS	2003–2016	11–14	Saalfeld and Lanctot (2017)
2	Herschel Island	BS, LL, SS, SB	1984–1986, 2007–2009	24	Grabowski et al. (2013)
3	Karrak Lake	SG, RG	1992–2014	23	Ross et al. (2017)
4	Coats Island	TM	1988–2007	20	Gaston et al. (2009)
5	Akimiski Island	CG	1993–2010	18	Brook et al. (2015)
6	Bylot Island	SG	1989–2012	24	Gauthier et al. (2013)
7	Zackenberg	SA	1996–2013	18	Reneerkens et al. (2016)
8	Carmarthenshire and Powys	WW	1982–1984, 2009–2011	30	Mallord et al. (2016)
9	Wytham	GT	1961–2007	47	Charmantier et al. (2008)
10	Antwerp	BT, GT	1979–2007	29	Matthysen et al. (2011)
11	Hoge Veluwe	PF	1973–2010	38	Both and Visser (2001), Lof et al. (2012)
11	Hoge Veluwe	GT, BT, CT, SP	1985–2005	20–21	Both et al. (2009)
12	Sindal and Hjorring	SP	1977–1997	21	Nielsen and Møller (2006)
13	Varald State Forest	CA, BlG	1979–2016	38	Wegge and Rolstad (2017)
14	Vranovice	CF, GT	1961–2007	47	Bauer et al. (2010)
14	Lednice	CF, GT	1961–2007	47	Bauer et al. (2010)
14	Lanzhot	CF, GT	1961–2007	47	Bauer et al. (2010)
15	Oulu	BT, GT, WT	1996–2011	14–16	Vatka et al. (2011), Vatka et al. (2014)
16	South‐Eastern Taimyr	BtG	1992–2016	25	Rakhimberdiev et al. (2018)
17	Teuri Island	RA	1984, 1985, 1987, 1992–2006	23	Watanuki et al. (2009)

Note

Abbreviations: AGP, American Golden Plover; BlG, Black Grouse; BS, Baird's Sandpiper; BT, Blue Tit; BtG, Bar‐tailed Godwit; CA, Capercaillie; CG, Canada Goose; CT, Collared Flycatcher; DO, Long‐billed Dowitcher; DU, Dunlin; GT, Great Tit; LL, Lapland Longspur; PF, Pied Flycatcher; PS, Pectoral Sandpiper; RA, Rhinoceros Auklet; ReP, Red Phalarope; RG, Ross' Goose; Rnp, Red‐necked Phalarope; SA, Sanderling; SB, Snow Bunting; SG, Snow Goose; SP, Sparrowhawk; SS, Semipalmated Sandpiper; TM, Thick‐billed Murre; WT, Willow Tit; WW, Wood Warbler.

**FIGURE 2:**
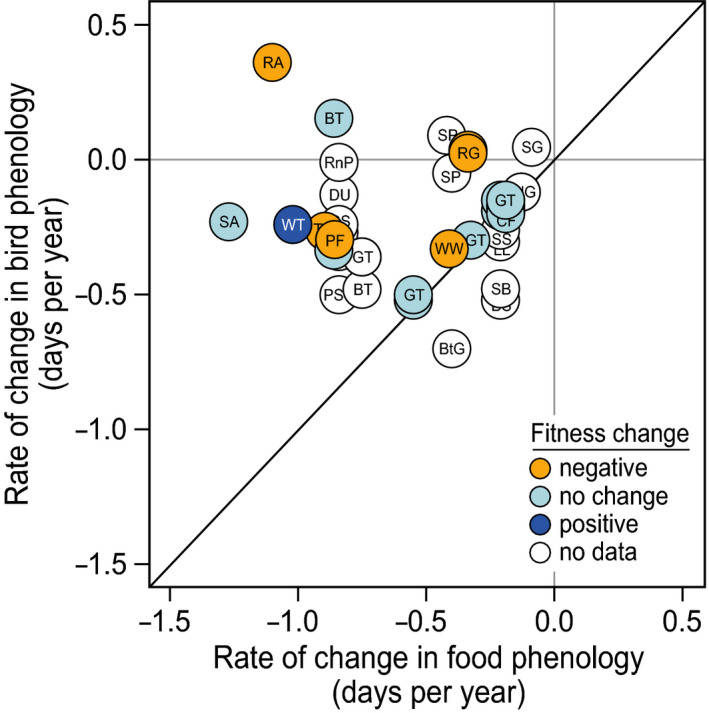
Rate of change in bird phenology plotted against rate of change in food phenology based on analyzed studies. Diagonal line indicates an equal rate of change for birds and their food. Colors indicate the change in fitness (measured or suggested) over the study period: orange—negative, light blue—no changes, dark blue—positive, white—data are not available. Abbreviations for the species are the same as in Table 1

The same abbreviations are used in Table 2 and Figure 2.

### 2.2 Analyzed components of selected studies

From each selected publication, we extracted the event describing food phenology (i.e., the yardstick), the event describing bird phenology, and the rate of shifts (in days per year) in both phenological events from the selected papers (i.e., negative in case of an advancement). We extracted both significant and non‐significant estimates from these papers and included an extra binary variable indicating whether each extracted estimate was reported significant. We calculated the rate of change in asynchrony, measured as number of days per year, by subtracting rate of food phenological shift from rate of bird phenological shift, which is positive when food phenology was advancing faster than bird phenology. For one paper, we used the reported trend of change in asynchrony directly because separate estimates for bird and food were not provided (Brook et al., 2015).

From each selected paper, we extracted breeding latitude. To standardize our measurements, we extracted other species‐specific data on migratory behavior (resident or migrant) and life‐history traits (body mass, clutch size, incubation duration) from the “Handbook of the Birds of the World” (del Hoyo et al., 1992–2011). If body mass was given as a range, we took the average, also if this range represented both males and females in sexually dimorphic species. Similarly, we extracted average clutch size and incubation duration from the published ranges. We divided birds into residents and migrants based on a coarse estimate of migration distance derived from the maps in (del Hoyo et al., 1992–2011). Partial migrants were included in the resident category. We also included the taxonomic level “order” to allow corrections for relatedness between species. A summary of collected data is presented in Table 2. Originally, we aimed at including an estimate of local temperature change to be used as a covariate, see for example, (Both et al., 2004), but we decided to exclude it, because temperature was either directly or indirectly used as a measure of food phenology in many of the selected studies. Other explanatory variables of potential relevance, for example, diet type, marine/terrestrial ecosystem, or the frequency of second broods, could not be included due to limited sample size.

**TABLE 2:** List of phenological pairs and their parameters included in the analyses

Species	Order	Body mass (g)	Clutch size	Incubation (d)	Migratory behavior	Latitude (°N)	Study duration (y)	Yardstick	Method of measuring food	Rate of food shift (days per year)	Rate of bird shift (days per year)	Rate of change in asynchrony (days per year)	Change in reproductive success *over the study period*	Reference
AGP	C	158	4	26	M	71.3	11	PA	SC	−0.84	−0.31	0.53	NA	Saalfeld and Lanctot (2017)
BS	C	47.5	4	20	M	69.6	24	FA	SC	−0.21	−0.52	−0.31	NA	Grabowski et al. (2013)
BtG	C	370.5	4	20.5	M	72.8	25	FA	Obs.	−0.4	−0.7	−0.3	NA	Rakhimberdiev et al. (2018)
BT	P	11.1	10	14	R	65	14	PA	FFM	−0.86	0.15	1.01	NO*	Vatka et al. (2014)
BT	P	11.1	10	14	R	52	21	PA	FFM	−0.75	−0.48	0.27	NA	Both et al. (2009)
BT	P	11.1	10	14	R	51.3	29	PA	MT	−0.55	−0.52	0.03	NO*	Matthysen et al. (2011)
CG	An	4,291	5.5	27	M	53	18	PQ	NDVI	NA	NA	0.08	NA	Brook et al. (2015)
CA	G	2,975	7.5	26	R	60.2	38	FA	GDD	−0.13	−0.12	0.01	NA	Wegge and Rolstad (2017)
CT	P	9.6	9	15	R	52	21	PA	FFM	−0.75	−0.36	0.39	NA	Both et al. (2009)
CF	P	12	6	13	M	48.7	47	other	FFM	−0.19	−0.2	0.01	NO	Bauer et al. (2010)
CF	P	12	6	13	M	48.8	47	other	FFM	−0.19	−0.19	0	NO	Bauer et al. (2010)
CF	P	12	6	13	M	48.9	47	other	FFM	−0.21	−0.19	0.02	NO	Bauer et al. (2010)
DU	C	59	4	22	M	71.3	14	PA	SC	−0.84	−0.13	0.71	NA	Saalfeld and Lanctot (2017)
BlG	G	1,050	8	26	R	60.2	38	FA	GDD	−0.13	−0.12	0.01	NA	Wegge and Rolstad (2017)
GT	P	17	8.5	13.5	R	65	16	PA	FFM	−0.86	−0.34	0.53	NO*	Vatka et al. (2014)
GT	P	17	8.5	13.5	R	52	21	PA	FFM	−0.75	−0.36	0.39	NA	Both et al. (2009)
GT	P	17	8.5	13.5	R	51.3	29	PA	MT	−0.55	−0.5	0.05	NO*	Matthysen et al. (2011)
GT	P	17	8.5	13.5	R	51.8	47	PA	FFM	−0.33	−0.3	0.03	NO	Charmantier et al. (2008)
GT	P	17	8.5	13.5	R	48.8	47	other	FFM	−0.19	−0.17	0.02	NO	Bauer et al. (2010)
GT	P	17	8.5	13.5	R	48.7	47	other	FFM	−0.19	−0.15	0.04	NO	Bauer et al. (2010)
GT	P	17	8.5	13.5	R	48.9	47	other	FFM	−0.21	−0.15	0.06	NO	Bauer et al. (2010)
SG	An	2,900	4.5	24	M	73.2	24	PQ	NDVI	−0.09	0.05	0.14	NA	Gauthier et al. (2013)
LL	P	28.75	5	11.75	M	69.6	24	FA	SC	−0.21	−0.30	−0.09	NA	Grabowski et al. (2013)
SG	An	2,900	4.5	24	M	67.2	23	PQ	NDVI	−0.34	0.04	0.37	YES−	Ross et al. (2017)
DO	C	112.5	4	20.5	M	71.3	11	PA	SC	−0.84	−0.27	0.57	NA	Saalfeld and Lanctot (2017)
PS	C	74.75	4	22	M	71.3	14	PA	SC	−0.84	−0.5	0.34	NA	Saalfeld and Lanctot (2017)
PF	P	12	6	14	M	52	28	PA	FFM	−0.87	−0.3	0.57	NA	Both and Visser (2001), Visser et al. (2012)
ReP	C	55	4	19	M	71.3	14	PA	SC	−0.84	−0.34	0.5	NA	Saalfeld and Lanctot (2017)
RnP	C	34	4	19	M	71.3	11	PA	SC	−0.84	−0.01	0.83	NA	Saalfeld and Lanctot (2017)
RA	P	533	1	35	R	44.4	23	other	Obs.	−1.1	0.36	1.46	YES−*	Watanuki et al. (2009)
RG	An	1,428.5	4.5	21.5	M	67.2	23	PQ	NDVI	−0.34	0.02	0.36	YES−	Ross et al. (2017)
SA	C	71.5	4	28	M	74.5	18	PA	Tr.	−1.27	−0.23	1.07	NO*	Reneerkens et al. (2016)
SS	C	30.5	4	21	M	71.3	14	PA	SC	−0.84	−0.24	0.6	NA	Saalfeld and Lanctot (2017)
SS	C	30.5	4	21	M	69.6	24	FA	SC	−0.21	−0.27	−0.05	NA	Grabowski et al. (2013)
SB	P	37	5	12.5	M	69.6	24	FA	SC	−0.21	−0.48	−0.27	NA	Grabowski et al. (2013)
SP	Ac	153	4.5	33	R	52	20	PA	Tr.	−0.42	0.09	0.51	NA	Both et al. (2009)
SP	Ac	153	4.5	33	R	57.5	21	other	Tr.	−0.4	−0.05	0.33	NA	Nielsen and Møller (2006)
TM	C	945	1	32.5	M	62.5	20	other	IC	−0.9	−0.27	0.63	YES−*	Gaston et al. (2009)
WT	P	11.5	7	14	R	65	14	PA	FFM	−1.02	−0.24	0.78	YES+*	Vatka et al. (2011)
WW	P	10.7	6	13	M	52.2	30	PA	Tr.	−0.41	−0.33	0.08	NO	Mallord et al. (2016)

Note

Species: see abbreviations in Table 1. Order: C, Charadriiformes; P, Passeriformes; An, Anseriformes; G, Galliformes; Ac, Accipitriformes. If the study duration for birds and their food differed, we used the duration of the study on birds. Yardstick: FA, food appearance; PA, peak abundance; PQ, peak quality; other, other yardsticks. Method of measuring food: EVI, enhanced vegetation index; SC, measuring snow cover; Obs., visual observation; FFM, frass‐fall method; MT, mean temperature; NDVI, normalized difference vegetation index; GDD, growing degree days; Tr., trapping; IC, ice cover. The rate of change in asynchrony was calculated by subtracting the rate of food phenological shift from rate of bird phenological shift. When bird and food shift rates were not reported (NA), we directly extracted the reported shift in asynchrony instead. Change in reproductive success *over the study period*: NO, no change; YES−, negative change; YES+, positive change; suggested changes are marked with an asterisk; NA, changes were not reported. A more detailed table can be found in the online Dryad data repository.

We noted study duration and whether changes in reproductive success *over the study period* were reported or suggested by the authors (Table 2). Reproductive success correlates were reported as either number of nestlings, nestling mass at a given age, nestling growth rate, nestling survival rate, fledging success, number of fledged young, mass at fledging, number of fledglings recruited into the breeding population the following year, ratio of juveniles to adults, nest success, second brood probability, or a combination of multiple parameters. All studies were grouped into three categories, where change in reproductive success *over the study period* was estimated as negative, as positive, or no change was found (Table 2). Due to the large variation in parameters indicating reproductive success, and thus the lack of standardized effect‐size measures, we refrained from conducting statistical tests linking rate of change in synchrony with changes in reproductive success *over the study period*. Instead, fitness measures are shown in Figure 2 to allow for a visual assessment of the data.

### 2.3 Statistical analyses

Our analysis consists of two components. We tested (I) whether rate of change in food phenology, bird phenology, and asynchrony significantly differed from zero, and (II) whether rate of change in asynchrony (in days per year) between birds and their food could be explained by breeding latitude, migratory behavior, and/or species' life‐history traits (body mass, clutch size, incubation duration).

#### 2.3.1 Rates of phenological shifts in birds and their food

We used linear mixed‐effect models to analyze whether the rate of change (days per year) in bird phenology, food phenology, and asynchrony differed significantly from zero. We (a priori) selected our random‐effect structure to account for repeated measurements within the same study site, and for relatedness between species, by including a random intercept for the taxonomic level “order” nested within study site. Normality assumptions were checked visually. The default function “intervals” from R‐package nlme was used to extract approximate 95% confidence intervals for all model estimates (Pinheiro & Bates, 2000).

#### 2.3.2 Rate of change in asynchrony explained

We used linear mixed‐effect models to analyze which biological traits best explained variation in the rate of change in asynchrony between birds and their food. As introduced above, we selected the following predictor variables: “breeding latitude,” “migratory behavior,” “body mass,” “clutch size,” and “incubation duration.” We log‐transformed “body mass” to prevent high leverage of data points for species with a large body mass. In addition, we excluded one data point (Watanuki et al., 2009) due to a high leverage (Cook's distance = 2.99).

Pairwise correlations of all predictor variables indicated that (log) body mass and incubation duration were positively correlated (*r* = 0.753) and that incubation duration and clutch size were negatively correlated (*r* = −0.603). Variance inflation factors (VIF) of the model containing additive effects of all predictors indicated multi‐collinearity issues (VIF = 9.30). To overcome these problems, and reduce dimensionality within our dataset, we conducted a principal component analysis (PCA) on the following normalized life‐history variables: (log) body mass, clutch size, and incubation duration. The first principal component (PC1) accounted for 81.3% of the total variance among these variables and had a positive loading for clutch size (0.36), a strong negative loading for incubation duration (−0.85), and a negative loading for body mass (−0.39). An increase in PC1 thus corresponds to a larger clutch size, shorter incubation duration, and a lower body mass. Variance inflation factors no longer indicated any concerns with multi‐collinearity after we replaced the three separate life‐history variables by the new predictor variable PC1 (hereafter called “life‐history traits”) (VIF = 1.59) (Zuur et al., 2009).

Next, we constructed a set of 18 candidate mixed models including additive effects and all two‐way interactions of “breeding latitude,” “migratory behavior,” and “life‐history traits.” All continuous predictor variables were mean‐centered to ease interpretation of regression coefficients (Schielzeth, 2010). Models were compared using Akaike's information criterion corrected for small sample size (Burnham & Anderson, 2002). Hierarchically more complex models (i.e., nested models with additional parameters) within ΔAIC_C_ = 2 of the top‐supported model were not considered informative/competitive (Arnold, 2010). We used the same random structure as in analysis (I) above, which was based upon a priori selection. We detected heteroscedasticity issues in our models, but modeling of variance structures either did not resolve the issue or led to convergence errors. Confidence intervals were extracted in the same way as in analysis (I). We additionally used linear mixed‐effect models to perform a post hoc analysis for each component of the rate of change in asynchrony separately (i.e., for rates of change in food and bird phenology). In both analyses, we added an extra binary predictor variable (“significancy”) which indicates whether the rate of phenological change was reported to be significant (assuming *p* ≤ 0.05). If significancy of the rate was not reported in the paper from which data were extracted, the data point was excluded from the analysis. This resulted in the exclusion of six data points for the analysis of the shift in food phenology and of three data points for the analysis of the shift in bird phenology. First, we tested whether the rate of change in food phenology could be explained by latitude of the study site and by significancy of the reported rate. We additionally included the interaction between both predictors. All random structures including “study site” led to violations of model assumptions in this analysis. We therefore proceeded by only correcting for relatedness between species by including a random intercept for “order.” Second, we tested whether the rate of change in bird phenology could be explained by a bird's breeding latitude, migratory behavior, life‐history traits, and significance of the reported rate. We additionally included all two‐way interactions between predictor variables. We corrected for repeated measures, and for relatedness between species, using the same random structure as in analysis (I).

### 2.4 Limitations of analyses

Results of the analyses should be interpreted with care due to the limited sample size, unbalanced nature of the dataset, variability of used methodologies among studies, and the fact that some predictors are not completely independent of each other. Moreover, the assumption that the analyzed time trends are linear might not be valid and could thus bias our analysis. Importantly, we refrain from conducting a classical meta‐analysis approach due to the lack of reported variance around the extracted point estimates in most studies.

## 3 RESULTS

### 3.1 Rates of phenological shifts in birds and their food

Mean length of 39 analyzed time series (excluding one extreme outlier, see methods) was 25.8 years (95% CI: 21.9 to 29. 6). Breeding phenology of birds advanced by a mean rate of 0.24 days per year (95% CI: 0.15 to 0.32, *F*_1,19_ = 31.7, *p* < 0.001). The average rate of advance for the phenology of bird food resources was 0.47 days per year (95% CI: 0.30 to 0.63, *F*_1,19_ = 34.1, *p* < 0.001). Accordingly, asynchrony between birds and their food increased at a rate of 0.24 days per year (95% CI: 0.06 to 0.42, *F*_1,19_ = 7.7, *p* =.012), indicating that birds became on average progressively less synchronized (Figure 2).

### 3.2 Rate of change in asynchrony explained

**FIGURE 3:**
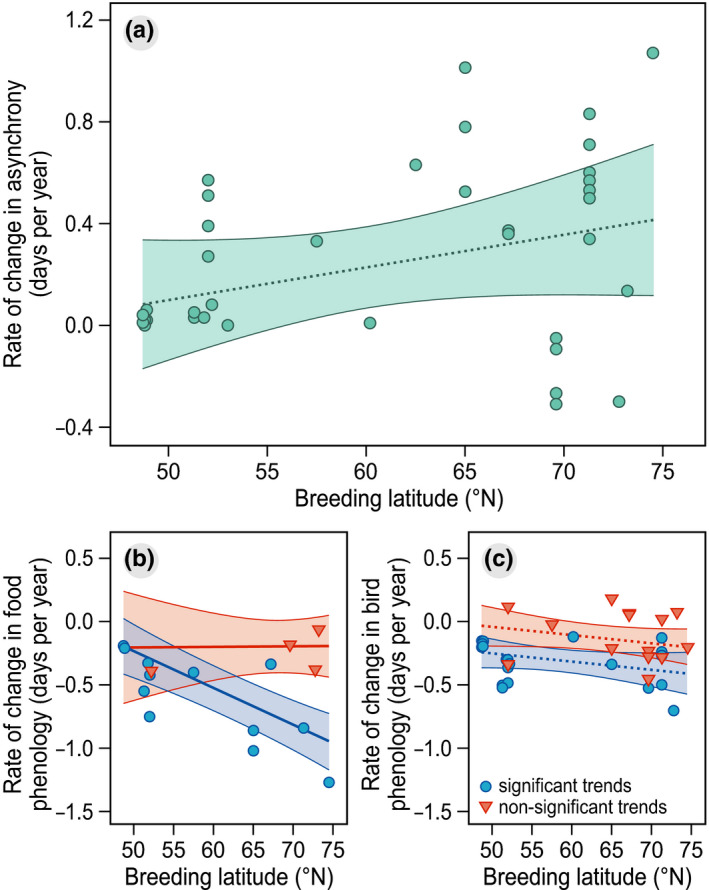
Rate of change in (a) asynchrony, (b) phenology of food, and (c) phenology of birds, plotted against breeding latitude. Model predictions are accompanied by 95% confidence intervals based on a *t*‐distribution, which are shown as shaded ribbons. Note that the effects of breeding latitude in (a) and (c) are not statistically significant and are therefore depicted by dotted lines

Our results did not provide support for an effect of any of the considered biological factors on the rate of change in asynchrony between birds and their food (Table 3). The model that indicated a positive effect of breeding latitude on the rate of change in synchrony (Figure 3a; model estimate 0.013, 95% CI: −0.004 to 0.030) did not outperform an intercept‐only model (ΔAIC_C_ = 0.321, Table 3).

**TABLE 3:** Overview of candidate mixed effects models about the rate of change in trophic asynchrony between birds and their main prey in long‐term studies

Model predictors	*k*	logLik	AIC_C_	ΔAIC_C_	*ω_i_*
**Intercept**	**4**	**1.21**	**6.76**	**0**	**0.287**
Latitude	5	2.37	7.08	0.321	0.244
Life history	5	1.74	8.34	1.574	0.131
Migration	5	1.33	9.16	2.400	0.086
Life history + latitude	6	2.59	9.44	2.676	0.075
Latitude + migration	6	2.37	9.88	3.112	0.061
Life history + migration	6	1.83	10.96	4.194	0.035
Life history + latitude + life history: latitude	7	2.78	12.06	5.296	0.020
Latitude + migration + latitude: migration	7	2.75	12.11	5.346	0.020
Life history + latitude + migration	7	2.60	12.41	5.648	0.017

Note

Only the 10 best models are shown. The following parameters are depicted: *k* – the number of parameters included in the model, logLik – the log‐likelihood, AIC_C_ – the Akaike Information criterion corrected for small sample size, ΔAIC_C_ – difference in AIC_C_ between the candidate model and the best model, *ω_i_* – model weights. The following model parameters are shown: “latitude” (i.e., breeding latitude), “migration” (i.e., migrant or resident), and “life history” (i.e., the first PC of the predictors “(log) body mass,” “incubation duration,” and “clutch size”). Note that all depicted models contain an identical random structure (i.e., a random intercept per order nested within study site). The top‐supported model is marked in bold.

However, the best model explaining shifts in food phenology indicated that significant shifts were faster at higher latitudes than at lower latitudes (ΔAIC_C_ = 5.091, Table S1; model estimate −0.029, 95% CI −0.039 to −0.019; Figure 3b), while nonsignificant shifts did not vary with latitude (Figure 3b; model estimate 0.000, 95% CI −0.020 to 0.021). In contrast, shifts in bird phenology did not vary with latitude, irrespective of whether they were reported as significant or not (Figure 3c). Instead, and as expected, shifts in bird phenology that were reported significant were faster than shifts that were reported nonsignificant (Table S2, model estimate −0.182, 95% CI: −0.296 to −0.068).

## 4 DISCUSSION

### 4.1 Which bird species are most prone to trophic mismatches?

Breeding phenology of birds advanced at a slower rate than the phenology of their food supply (Figure 2), suggesting that birds are experiencing an increasingly large trophic mismatch, which corresponds with earlier findings (Visser et al., 2012). We found that shifts in food phenology were faster at higher latitudes, but only for trends that were reported significant (Figure 3b), while shifts in bird phenology did not show such a pattern (Figure 3c). This would imply larger trophic mismatches at higher latitudes, although the positive trend that we found between rate of change in asynchrony and latitude was not significant (Figure 3a). Although this might partly be explained by a limited sample size, the lack of evidence was nevertheless surprising given findings of previous studies (Post et al., 2018). Temperatures in the Arctic have increased two to three times as fast as the global average (IPCC, 2014

), which suggests birds breeding there could be more vulnerable to trophic mismatches. Arctic ecosystems are considered to be more sensitive to climate warming than ecosystems at lower latitudes (Høye et al., 2007), in which lower trophic levels seem able to keep pace with rapid changes (Lameris, Jochems, et al., 2017). Birds, on the other hand, may not be able to advance reproductive phenology at the same rate. They seem hampered in adjusting the timing of spring migration (Knudsen et al., 2011), as they may lack reliable cues to advance migration in pace with earlier food phenology (Kölzsch et al., 2015), or as they may be constrained in time or resources (Schmaljohann & Both, 2017). Alternatively, advancements in breeding phenology may be maladaptive and come with reductions in adult survival or an increased risk of clutch predation (Lof et al., 2012; Reneerkens et al., 2016; Visser et al., 2012).

Our results should be interpreted with care because (a) effects of breeding latitude are intertwined with effects of spring temperature change and study duration (Figure S1, Methods S1), also see (Post et al., 2018). (b) In shorter studies, which more often took place at higher latitudes, the rate of change in asynchrony is more likely to be biased by extreme years. (c) An alternative, but not mutually exclusive, explanation could be the consequence of a possible publication bias: If only significant changes are published, this would require stronger effects in shorter studies. (d) Our analysis is limited by a small sample size and an unbalanced dataset, and thus, we were unable to include all potentially important life‐history traits, for example, food specialization, capital versus income breeders, precocial versus altricial birds. (e) Measures of food phenology and bird phenology might be biased by observer effort. (f) Various environmental indicators used for determining food phenology might differ in the precision with which they describe food availability.

Changes in reproductive success over time were reported for less than half of the inspected predator–prey interactions: In five cases, a negative change was claimed, in fourteen cases—no change, and for one case—a positive change. The most often reported fitness effects were nestling survival rates (seven out of twenty studies) and/or nestling mass or nestling growth rate (seven out of twenty studies). A visual assessment of these results indicated that a faster increase in asynchrony over time does not necessarily result in negative reproductive success (Figure 2). It is important to note that the absence of the change in reproductive success *over the study period* does not exclude the scenario that in particular years where birds are less synchronized with their food, they incurred lower success (Charmantier et al., 2008; Vatka et al., 2014). Moreover, a long‐term temporal trend can also be masked when there is large variation in effects from one to another year (Gauthier et al., 2013). Furthermore, some studies looked at average reproductive success for the population as a whole (Ross et al., 2017), whereas other studies considered the mismatch effects on fitness at the individual level (Charmantier et al., 2008; Reed et al., 2013). Further note that mismatches with the food peak for nestlings can be adaptive, through optimizing the trade‐off between costs and benefits (Visser et al., 2012) and need not necessarily arise from global warming (Perrins, 1965).

Studies not only differed in their methodology with which reproductive success was assessed, but also in measurements of shifts in phenology of bird reproduction and food availability. This complicates comparisons and, in some cases, may explain why our results do not match our expectations. Most striking is the large variation in the methods used to measure phenology of food abundance (i.e., the yardsticks, Table 2). Yardsticks should be ecologically relevant for the studied species. Below we discuss methodological issues regarding yardsticks and provide recommendations for determining the best possible yardsticks for studies on reproductive phenology.

### 4.2 Indicators of food availability

The term “yardstick” was first introduced by Visser and Both, who made a case for providing a measure that reflects how much the phenology of consumers should change to keep up with the warming‐induced changes in the environment (Visser & Both, 2005). The phenology of a consumer's food abundance was proposed as the most suitable yardstick (Visser & Both, 2005). Ideally, such a yardstick is a direct measure of available food (or, even better, energy intake) for the consumer's offspring. Conducting direct measurements of food availability has however rarely been done throughout the entire year (Ahmedou Salem et al., 2014). Instead, some indicator to measure food availability was often used. From the 39 time series included in the analysis, nineteen used food‐related indicators, for example, food abundance or quality, while twenty used large‐scale environmental indicators, for example, climatic variables or vegetation indexes extracted from remote‐sensing data. A qualitative comparison of the rate of change in food phenology between these two indicator groups did not indicate any differences.

Environmental indicators are typically extracted from remote‐sensing data, for example, the normalized difference vegetation index NDVI (Brook et al., 2015; Ross et al., 2017), or from climate data, for example, temperature (Reed et al., 2013; Visser et al., 1998), or a temperature sum such as growing degree days GDD (Clausen & Clausen, 2013; Wegge & Rolstad, 2017), which allow analyzing long time series for almost any location worldwide. Nevertheless, such indicators should be used with care when quantifying phenological mismatches. The assumption that environmental indicators are a reliable measure for the phenology of food availability is often based on correlations with phenology of local food abundance. This may be problematic. Firstly, not all studies provide support for these correlations with data from ground‐based studies, and studies which are used as support are sometimes based on data for different study species or locations, and/or cover a small number of years. Secondly, environmental indicators are often “2nd degree indicators,” as they correlate with food abundance, which then should correlate with the actual food availability. Only if the strength of the correlation is sufficiently large, its proxy will have a good predictive power. In fact, they can be even “3rd degree indicators.” For example, NDVI is an indirect, remote measure of the greening up of vegetation (3rd degree), which is in some cases used as an indicator for the emergence of insect larvae within the vegetation (food abundance, 2nd degree), which in turn indicates insect availability (1st degree) (Pettorelli et al., 2011). While using large‐scale environmental indicators may allow for analyzing long time series, errors will accumulate at each indirect step, and therefore, they may not be an optimal yardstick to analyze phenological changes in the light of climate change. Thirdly, the relationship between a food source and its indicator can be nonlinear (Iler et al., 2013). Ignoring this nonlinearity could result in under‐ or overestimation of the actual mismatch.

Direct measures of food abundance can provide a much closer indicator for food availability for the consumer in phenological studies. Methods used in the studies we analyzed included, for example, frass net contents, indicating the abundance of caterpillars under a few representative trees in a study site, for example, (Both et al., 2009), or the contents of pitfalls, indicating the abundance of (mostly) ground‐dwelling arthropods, for example, (Reneerkens et al., 2016; Tulp & Schekkerman, 2008). In herbivores, for which quality of food rather than quantity is often a more important driver of available energy for the offspring, some studies measured protein concentration in forage plants (van der Graaf et al., 2006). Also, fulfilling nutritional requirements by eating specific prey items might be important for normal early development of chicks in insectivorous birds (Samplonius et al., 2016). However, a thorough understanding of food availability can only be achieved when the study species' diet is known in detail, and under varying ecological conditions, and prey are sampled using appropriate methods (Zwarts et al., 1992). Detailed knowledge on the consumers' diet can, for example, be obtained using DNA barcoding (Symondson, 2002; Valentini et al., 2009; Wirta et al., 2015). This should concentrate on variation within and between years to see how differences in food availability result in consumed diet, and its consequences for reproductive success. As far as we know, no studies have specifically connected within‐ and between‐year variation in food abundance/phenology, diet choice, and reproductive success, although several studies have attempted this (Naef‐Daenzer et al., 2000; Samplonius et al., 2016).

### 4.3 What is the right yardstick?

**FIGURE 4:**
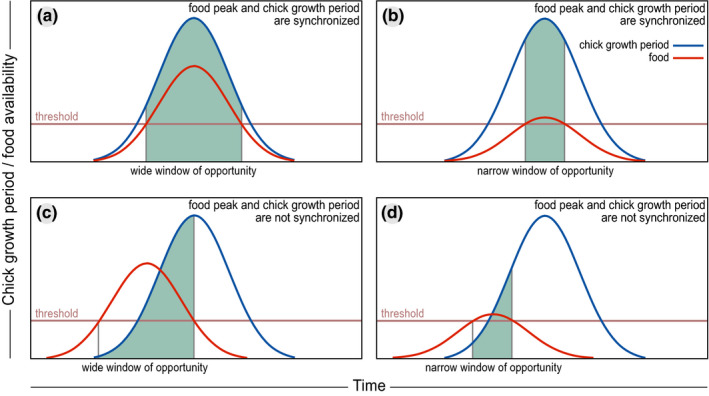
Conceptual figure indicating when trophic mismatches may lead to reductions in fitness. Chicks (blue line) as well as their food (red line) show a peaked occurrence throughout the season. Chicks are mismatched when food level is below their threshold requirement (horizontal line). The period of match is indicated by green shading. (a) Large synchrony and a high food peak result in the highest degree of match. (b) Despite synchronization with the food peak, a large part of the chick population is mismatched when the food peak is low. (c) Even when chicks and food are not synchronized, the proportion of mismatched chicks is low when the food peak is high. (d) Extreme degree of mismatch, due to the combination of an asynchronous and low food peak

The timing of the food peak was suggested as a universal yardstick to describe fitness consequences of trophic mismatches between avian consumers and their prey (Visser & Both, 2005), although theoretical studies showed that to study fitness effects, it is important to consider the entire period during which food abundance sufficiently meets and overlaps with the food requirements of the offspring throughout the breeding season (Durant et al., 2005, 2007). Several empirical studies indeed indicate that asynchrony with a food peak may not lead to fitness consequences when food is sufficiently abundant during the entire season (Corkery et al., 2019; Dunn et al., 2011; Reneerkens et al., 2016; Wesołowski & Rowiński, 2014). Vice versa, a good temporal match between offspring growth period and food peak might still result in negative fitness consequences when food availability during the entire season is low (Vatka et al., 2014). (Figure 4).

Whether the peak in food availability will be the best yardstick to describe a trophic mismatch will depend on the shape of the seasonal food availability curve. Using the timing of the food peak as a yardstick to measure the extent of a phenological mismatch will only be relevant in environments where food availability shows a pronounced seasonal peak (Doiron et al., 2014; Lameris, Scholten, et al., 2017; Richman et al., 2015; Visser & Both, 2005). In these cases, the timing of the food peak can be a better functioning yardstick compared to more complicated measures such as temporal overlap between phenological distributions of resource and consumer (Ramakers et al., 2019).

In systems without a single pronounced peak in food availability, however, food availability varies from day to day (Figure S2a), which might be attributable to local weather conditions. Several consecutive days with adverse weather and reduced food availability can severely reduce growth rates and survival probabilities of precocial chicks (Schekkerman et al., 1998; Tulp & Schekkerman, 2008). In this case, defining a period of sufficient food availability, above a minimum threshold that is required for offspring growth and survival (Figure S2b) (Saalfeld et al., 2019; Vatka et al., 2014), may be a more relevant yardstick for defining a mismatch than a food peak.

Variation in food availability and abundance may also come about by variation in timing of different food items. A species may profit from multiple “peaks” in food availability if it preys on species which show a sequence in their timing of emergence (Vatka et al., 2016). Therefore, decomposing food availability into separately studied elements can provide relevant details that may help understand the phenology of food abundance better, if we know how diets depend on food availability (Figure S2c).

Identifying and measuring the relevant yardstick, in relation to which reproductive phenology and potential fitness consequences should be studied, is a challenging task. Defining a period of sufficient food for growth on the basis of a study‐specific threshold can be a promising way forward. Such thresholds can be estimated by analyzing when and under what conditions of food availability reductions in growth or survival occur (Schekkerman et al., 2003), or by reconstructing energy budgets in relation to food availability to make inferences about minimum food thresholds (van Gils et al., 2004; Piersma et al., 1995; Wiersma & Piersma, 1994). Thresholds could vary with both internal (e.g., age, sex, body size) and/or external (e.g., weather variables) factors. Demand curves based on metabolic rates of chicks could thus be a significant step forward in understanding how mismatches translate to effects at the levels of individuals and populations (Kwon et al., 2019). However, more sophisticated methods to quantify mismatches might not always result in a better understanding of demographic parameters (Ramakers et al., 2019). We suggest that an ideal approach to study phenological mismatches would include several consecutive steps: (a) clarifying the strength of interactions between consumers and resources by a rigorous diet analysis and quantifying dietary specialization of offspring (e.g., DNA analysis of feces or observations of food intake), (b) determining whether food abundance or quality is a limiting factor for offspring growth and survival, (c) defining the most reliable indicator for relevant food items, if necessary, (d) simultaneously measuring food phenology, bird phenology and correlates of bird reproductive success, (e) estimating the threshold for the occurrence of fitness effects or a demand curve, (f) estimating the effect of mismatch based on both synchronization of bird phenology with the food peak and the degree of overlap with the window of opportunity. Besides this, the match–mismatch hypothesis should be tested along with alternative hypotheses explaining fitness effects in the context of intra‐ and interspecific interactions (e.g., density and time dependence, top‐down effects, size‐mediated priority effects) (Johansson et al., 2015; Kharouba & Wolkovich, 2020).

## 5 CONCLUSION

Our analyses showed no obvious general factors explaining the occurrence of phenological mismatches, although food phenology advanced faster at higher latitudes for trends that were reported as significant, while changes in bird phenology did not vary with latitude. We suggest that, besides the limited sample size of the dataset, this is at least partly due to the various yardsticks used to describe food phenology. We plea for the use of yardsticks that are ecologically relevant in order to assess whether increasing asynchrony results in fitness consequences. Long‐term studies that incorporate standardized methodologies are needed to assess changes in reproductive success over time and improve insights and predictions regarding which bird species are most likely to experience trophic mismatch with global warming, and how individuals and populations will respond.

## CONFLICT OF INTEREST

We declare that we have no competing interests.

## AUTHOR CONTRIBUTION

**Mikhail K. Zhemchuzhnikov:** Conceptualization (equal); formal analysis (supporting); visualization (equal); writing – original draft (lead); writing – review and editing (equal). **Tom S. L. Versluijs:** Conceptualization (equal); formal analysis (lead); visualization (equal); writing – review and editing (equal). **Thomas K. Lameris:** Conceptualization (equal); visualization (equal); writing – review and editing (equal). **Jeroen Reneerkens:** Conceptualization (equal); formal analysis (supporting); writing – review and editing (equal). **Christiaan Both:** Formal analysis (supporting); writing – review and editing (equal). **Jan A. van Gils:** Conceptualization (equal); funding acquisition (lead); supervision (lead); writing – review and editing (equal).

## Supporting information

Supplementary Material

## Data Availability

Data and R‐scripts are available in Dryad at https://doi.org/10.5061/dryad.547d7wm7m.
