# Supplementary material for: Exploring the drivers of variation in trophic mismatches: A systematic review of long‐term avian studies
Source: Ecol Evol. 2021 Mar 20;11(9):3710–25. doi: 10.1002/ece3.7346 (PMC8093693; doi:10.1002/ece3.7346)
Supplement: Supplementary file 1 — Supplementary Material [file ECE3-11-3710-s001.docx]

**Box S1.** Glossary with key definitions.

| Term | Definition | Reference |
| --- | --- | --- |
| Food peak | The highest availability or quality of food. The timing of the food peak is often expressed as median date of highest food availability within a season. |  |
| Phenological mismatch (or phenological asynchrony) | Disruption of interaction between species caused by different rates of time shift in regularly repeated phases in their life cycles. | (Renner & Zohner, 2018) |
| Threshold | Minimally required food available for consumers to meet sufficient energy and/or nutrients to realize growth without fitness consequences. | (Schekkerman, Tulp, Piersma, & Visser, 2003) |
| Trophic mismatch | Phenological mismatch between consumer and food source in the context of trophic interactions. |  |
| Window of opportunity | Period during which food conditions are sufficient to satisfy the energetic requirements of the offspring. | (Tulp, 2007) |

**Table S1.** Overview of candidate mixed effects models about the rate of change in food phenology in long-term studies. The following parameters are depicted: *k* – the number of parameters included in the model, logLik – the log-likelihood, AIC_C_ – the Akaike Information criterion corrected for small sample size, ΔAIC_C_ – difference in AIC_C_ between the candidate model and the best model, ω*_i_* – model weights. The following model parameters are shown: ‘latitude’ (i.e. breeding latitude) and ‘significancy’ (i.e. significant or non-significant trend). Note that all depicted models contain an identical random structure (random intercept per order). The top-supported model is marked in bold.

| Model predictors | *k* | logLik | AIC_C_ | ΔAIC_C_ | ω*_i_* |
| --- | --- | --- | --- | --- | --- |
| **latitude:significancy** | **6** | **9.77** | **-4.31** | **0** | **0.927** |
| latitude + significancy | 5 | 5.72 | 0.78 | 5.091 | 0.073 |
| significancy | 4 | -2.47 | 14.37 | 18.673 | 0 |
| latitude | 4 | -5.86 | 21.14 | 25.447 | 0 |
| intercept | 3 | -7.77 | 22.38 | 26.682 | 0 |

**Table S2.** Overview of candidate mixed effects models about the rate of change in bird phenology in long-term studies. Only the 10 best models are shown. The following parameters are depicted: *k* – the number of parameters included in the model, logLik – the log-likelihood, AIC_C_ – the Akaike Information criterion corrected for small sample size, ΔAIC_C_ – difference in AIC_C_ between the candidate model and the best model, ω*_i_* – model weights. The following model parameters are shown: ‘latitude’ (i.e. breeding latitude), ‘migration’ (i.e. migrant or resident), ‘life history’ (i.e. the first PC of the predictors ‘(log) body mass,’ ‘incubation duration’ and ‘clutch size’) and ‘significancy’ (i.e. significant or non-significant ternd). Note that all depicted models contain an identical random structure (i.e. random intercept per order nested within study site). The top-supported model is marked in bold.

| Model predictors | *k* | logLik | AIC_C_ | ΔAIC_C_ | ω*_i_* |
| --- | --- | --- | --- | --- | --- |
| **significancy** | **5** | **14.44** | **-16.87** | **0** | **0.239** |
| life history + latitude + significancy | 7 | 17.38 | -16.76 | 0.115 | 0.226 |
| latitude + significancy | 6 | 15.55 | -16.2 | 0.668 | 0.171 |
| life history + significancy | 6 | 14.93 | -14.96 | 1.91 | 0.092 |
| migration + significancy | 6 | 14.6 | -14.31 | 2.56 | 0.066 |
| life history + latitude + migration + significancy | 8 | 17.38 | -13.43 | 3.444 | 0.043 |
| latitude + migration + significancy | 7 | 15.55 | -13.1 | 3.771 | 0.036 |
| life history + migration + significancy | 7 | 15.26 | -12.53 | 4.345 | 0.027 |
| life history + latitude + migration + significancy + latitude:migration | 9 | 18.18 | -11.43 | 5.445 | 0.016 |
| life history + latitude + migration + significancy + life history:latitude | 9 | 17.94 | -10.95 | 5.919 | 0.012 |

**Methods S1.** We used the R package “RNCEP” to extract study site-specific daily surface temperatures at noon (‘air.sig995’) from the NCEP Reanalysis-I data set, for a three month spring window spanning the duration of each study (Kemp, Emiel van Loon, Shamoun-Baranes, & Bouten, 2012). The considered spring window was species-specific as it spanned the period from one month before, until two months after the start of each species’ breeding season. Because not all studies specified the start of the breeding season for each species, we instead opted for a standardized approach for all studies and extracted species-specific data on the start of the breeding season from the Handbook of the Birds of the World (Del Hoyo, Elliott, Sargatal, & Christie, 2016). We then fitted a separate linear regression through the collected temperature data for the period of the study for each study site and used the slope of this regression as a measure of study and species-specific temperature change in degrees per decade. However, because temperature was either directly or indirectly used as a measure of food phenology in many of the selected studies, we decided to exclude the local rate of change in spring temperature as a predictor variable in our models.

**
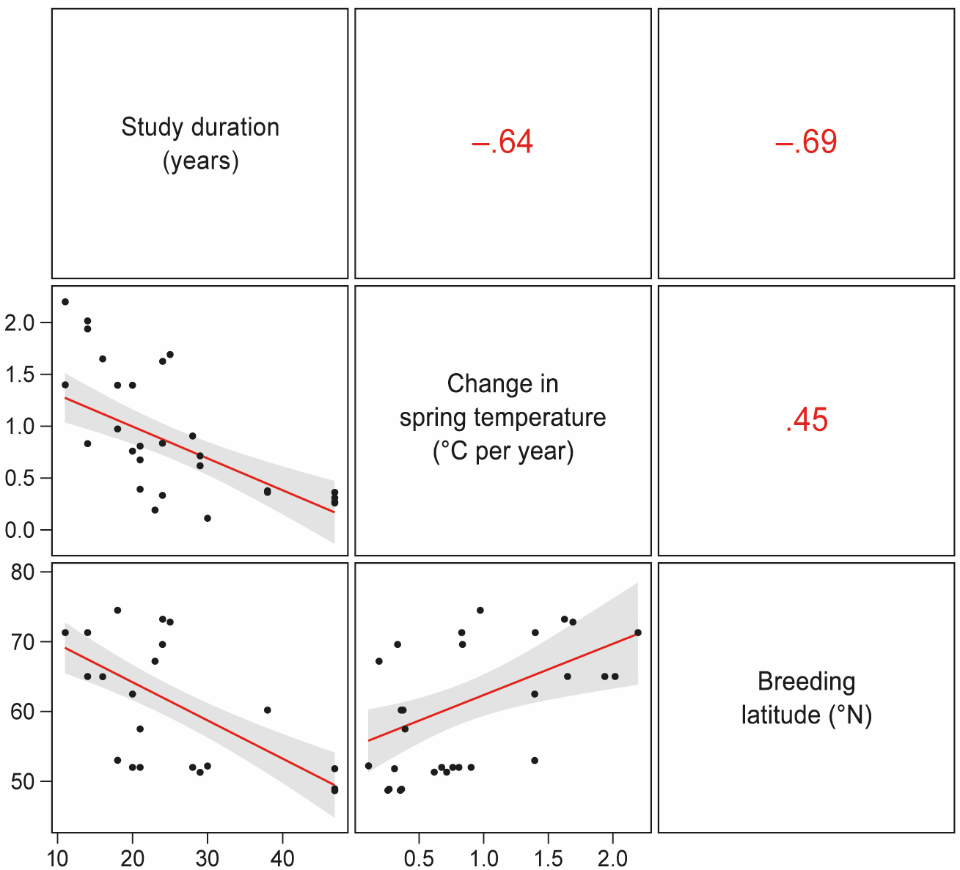
**

**Figure S1.** Correlations between study duration, change in local spring temperature, and breeding latitude. Dots represent raw data points. Red lines represent simple linear regressions with 95% confidence intervals depicted as grey bands. Red numbers are Pearson correlation coefficients.

**
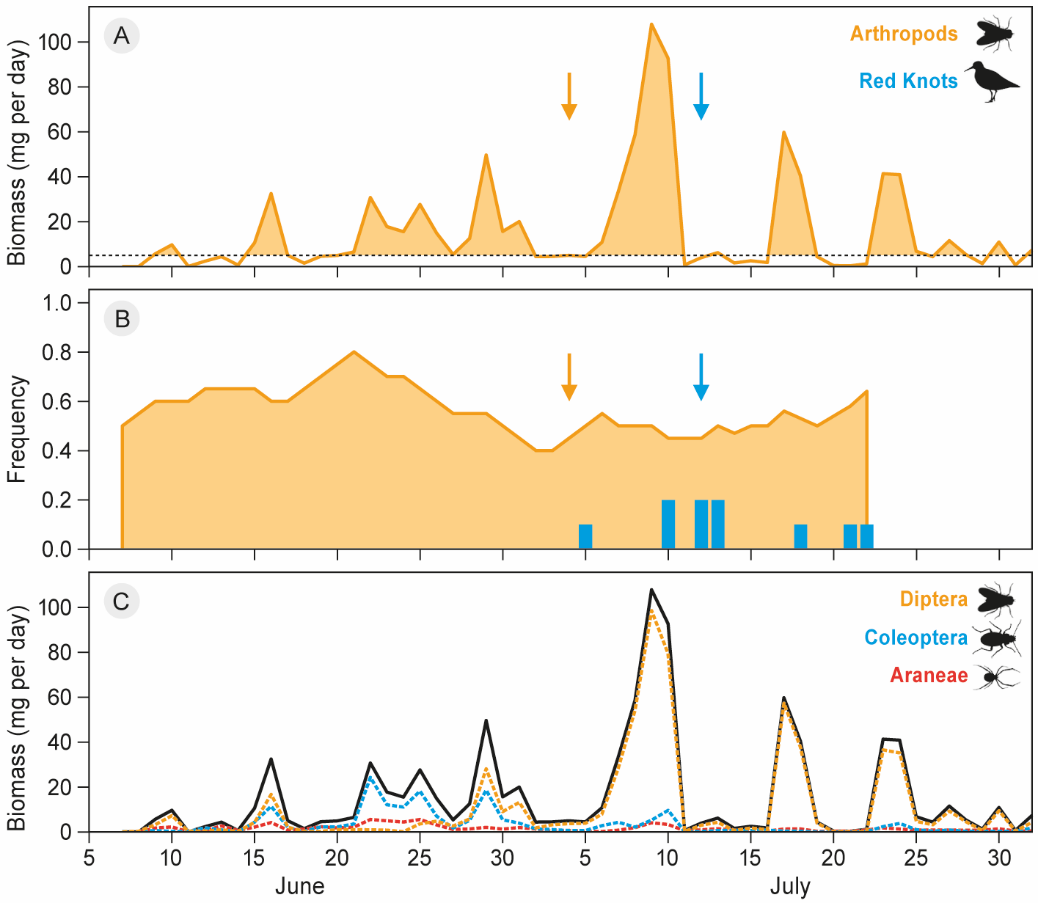
**

**Figure S2**. (A) Dynamics of arthropod biomass (mg per day) in the pitfall traps across the summer season of 2018 at our study site in northern Taimyr, Russia. The horizontal dashed line indicates the threshold of 5 mg per day, above which chicks of red knots are assumed to grow (Schekkerman et al., 2003). Seasonal peak of arthropods, measured as the median date of arthropod abundance (Reneerkens et al., 2016), is depicted by the orange arrow. Mean red knot *Calidris canutus* hatch date (n=10 clutches) is depicted by the blue arrow. For red knots breeding in the Russian Arctic, we found that their median hatch date occurred several days after the peak date in food availability (Lameris, Zhemchuzhnikov, ten Horn, & van Gils, unpublished data). (B) When we compared the number of days with food above the threshold of 5 mg/day (Schekkerman et al., 1998), there was no difference in the number of days with sufficient food for chicks that hatched in synchrony with the food peak and chicks that hatched later. The proportion of days with arthropod biomass > 5 mg per day for a 10-day running mean period (starting on June 5) is depicted by the orange polygon. Red knot hatch dates of individual clutches are shown by blue bars. The arrows are equivalent to those described under (A). (C) Total arthropod biomass (black line) split up into the following taxonomical groups: flies and mosquitos (Diptera; orange line), beetles (Coleoptera; blue line), and spiders (Araneae; red line). Peaks in arthropod food availability for red knot chicks vary over the season: Early in the season, mostly spider species (Araneae) and beetles (Coleoptera) are abundant, while peaks later in the season almost solely consist of species of flies (Diptera). Food abundance in most cases is the sum of different taxonomic groups that can respond differently to environmental factors (Kankaanpää et al., 2018; Koltz, Schmidt, & Høye, 2018). Prey species are thus likely to respond differently to climate warming, which might change the shape of the food availability curve with important consequences for consumers (Schmidt et al., 2017).

References:

del Hoyo, J., Elliott, A., Sargatal, J., & Christie, D. (1992–2013). Handbook of the Birds of the World. *Lynx Edicions, Barcelona, Spain*.

Kankaanpää, T., Skov, K., Abrego, N., Lund, M., Schmidt, N. M., & Roslin, T. (2018). Spatiotemporal snowmelt patterns within a high Arctic landscape, with implications for flora and fauna. *Arctic, Antarctic, and Alpine Research*, *50*(1), 1–17. doi: 10.1080/15230430.2017.1415624

Kemp, M. U., Emiel van Loon, E., Shamoun-Baranes, J., & Bouten, W. (2012). RNCEP: Global weather and climate data at your fingertips. *Methods in Ecology and Evolution*, *3*(1), 65–70. doi: 10.1111/j.2041-210X.2011.00138.x

Koltz, A. M., Schmidt, N. M., & Høye, T. T. (2018). Differential arthropod responses to warming are altering the structure of arctic communities. *Royal Society Open Science*, *5*(4), :171503. doi: 10.1098/rsos.171503

Reneerkens, J., Schmidt, N. M., Gilg, O., Hansen, J., Hansen, L. H., Moreau, J., & Piersma, T. (2016). Effects of food abundance and early clutch predation on reproductive timing in a high Arctic shorebird exposed to advancements in arthropod abundance. *Ecology and Evolution*, *6*(20), 7375–7386. doi: 10.1002/ece3.2361

Renner, S. S., & Zohner, C. M. (2018). Climate change and phenological mismatch in trophic interactions among plants, insects and vertebrates. *Annual Review of Ecology, Evolution, and Systematics*, *49*(1), 165–182. doi: 10.1146/annurev-ecolsys-110617-062535

Schekkerman, H., Tulp, I., Piersma, T., & Visser, G. H. (2003). Mechanisms promoting higher growth rate in arctic than in temperate shorebirds. *Oecologia*, *134*(3), 332–342. doi: 10.1007/s00442-002-1124-0

Schekkerman, H., Van Roomen, M. W. J., & Underhill, L. G. (1998). Growth, behaviour of broods and weather-related variation in breeding productivity of Curlew Sandpipers Calidris ferruginea. *Ardea*, *86*(2), 153–168.

Schmidt, N. M., Hardwick, B., Gilg, O., Høye, T. T., Krogh, P. H., Meltofte, H., … Roslin, T. (2017). Interaction webs in arctic ecosystems: Determinants of arctic change? *Ambio*, *46*(s1), 12–25. doi: 10.1007/s13280-016-0862-x

Tulp, I. (2007). The arctic pulse: timing of breeding in long-distance migrant shorebirds.
